# Gleanings in Science and Medicine

**Published:** 1893-04-08

**Authors:** 


### Gleanings in Science and Medicine.

At certain specified hospitals at Calcutta, a selected committee of doctors have been ordered by the Government of India to undertake an exhaustive enquiry into a new method of treating snake bites by injections of strychnine. In Australia the method is said to have already been proved a definite cure; and in three cases, where the application was proved beneficial, the bites had been considered of a atal nature.

It would be well if the Government Boards could educate their financial officials as carefully and systematicelly as the School Board does its teachers. We should then observe less astonishing discrepancies in the cost of commodities in the Government establishments. Statistics show that where in one district a workhouse is being provided with candles at 1½d. the pound, in another district the union is charged 8d. The one price is as unreasonably low as the other is unreasonably high, and both charges indicate that inquiry is necessary. Unfortunately, those in power know but little of the details of management, and for this reason a marked improvement would be effected were women appointed on the Board to enquire into household management.

We hear from Mannheim that the German tailor who has just put forward a claim to the invention of a bullet-proof material which can be used either as coat or shield has been ordered to appear before the German Emperor. Should Herr Dowes' discovery prove efficacious, as it claims to be, it seems that it may almost revolutionise military tactics as they now exist; but German newspapers do not hint even at this possibility—in several cases the fact alone is mentioned, and passed over without comment, for Herr Dowes is not the first claimant of the invention. In the Duke of Wellington's time an anecdote is told how one day a stranger was ushered into his presence and, having submitted a bullet-proof jacket for the great warrior's inspection, made a request that it should be introduced among his soldiers. "All right," the Duke answered, "Put it on," and then, ringing the bell, he told his aide-de-camp to order two soldiers to appear with loaded rifles, at which omninous words, it is said, the inventor quickly retired from the presence of his would-be patron.

It is always humiliating to our pride to discover that our swans are geese. We have boasted for some years now, as a nation, of the luxury of our afternoon cup of tea. We are now discovering that much of what we consume as such is not "tea" itself, but just a mixture of such heterogeneous leaves as the sloe, the poplar, the willow, and the oak, and the adulteration does not stop here; a chemical coating transforms these into so-called "black" tea, and another chemical preparation into so-called "green" tea; we do not say that the tea leaf itself is entirely excluded, but its occurrence amongst the others is rare. One has only to place a small quantity of the leaves under a magnifying glass to see how diverse are their forms, and to note the absence of the closely serrated form of the leaf after which the compound takes its name. So long as the public demand commodities at abnormally low prices so long must we foresee that vendors of the same must discover some way of meeting the difficulty; and it is only of the low-priced pound of tea that we are speaking now; and from which we ought barely, perhaps, to expect a happy result after too close an analysis.

